# The Effect of *Cupressus Sempervirens* on Ulcerative Colitis: Do Pathological Changes Improve by Oxidative Stress Amelioration?

**DOI:** 10.31661/gmj.v8i0.1650

**Published:** 2019-12-31

**Authors:** Nastaran Samimi, Mojtaba Farjam

**Affiliations:** ^1^Noncommunicable Diseases Research Center, Fasa University of Medical Sciences, Fasa, Iran; ^2^Student Research Committee, Fasa University of Medical Sciences, Fasa, Iran

**Keywords:** Ulcerative Colitis, Inflammatory Bowel Diseases, Oxidative Stress, Regeneration, Histopathology

## Dear Editor,

An important paradigm in the biochemical basis of diseases and pharmacological approach during recent decades has been focused on oxidative stress modulation in many inflammatory diseases. Ulcerative colitis (UC) has attracted great attention for the past few years because of the wait for better therapeutic modalities. As many other inflammatory diseases, this disease is also believed to be partially the result of extensive oxidative stress [1]. There are many review articles and many more original articles that have emphasized on oxidative stress as an important factor for the pathogenesis of ulcerative colitis [2-4]. Therefore, researchers have tried many anti-oxidative therapies to treat UC not only in animal models but also in human disease [5, 6]. Unexpectedly, the decrease in important factors pertinent to oxidative stress have not been constantly related to restoration of colon structure in accordance with pathological studies [7]. In human disease, there have been some exceptions to provide accurate evidences suggesting that oxidative stress is a pivotal prospect in the modulation of the destruction of colon tissue. In our experimental study, we used *Cupressus sempervirens* to treat UC in the rat model that published the resulting data of the study in the Journal of Coloproctology recently [8]. Although our treatment was shown to be effective in the modulation of all aspects of oxidative stress factors and reduction of inflammation, we did not see the effective restoration of tissue of the colon. Our finding is the first report of the use of this herbal medication as a therapy for UC model, and although *C. sempervirens* have shown effective anti-oxidative characteristics, this effect has not been fully fruitful in the restoration of normal colon histology. There are many possible explanations, such as short duration of studies while the action of the medication is slow and the variation of histopathological scoring systems of UC. Moreover, we believe that although oxidative stress is a very important step in the pathogenesis of UC, nevertheless this is not the factor that its modulation can inevitably result in the tissue restoration in this disease. To restore injured tissue and reach optimal treatment, incorporation of immunomodulation with anti-oxidant agents is very important. Hereby, we ask you to publish this letter and send it to scientists working on oxidative stress to open a window for debate on the real effect of oxidative stress on the pathological findings in UC and also the modulation of this type of stress in the restoration of the normal structure of colon tissue.

## Conflict of Interest

The authors declare that there is no conflict of interest.

## Acknowledgment

We acknowledge Fasa University of Medical Sciences for supporting our chain studies on immunopharmacology of UC.
